# The synthesis and characterization of polyorganosiloxane nanoparticles from 3-mercaptopropyltrimethoxysilane for preparation of nanocomposite films via photoinitiated thiol-ene polymerization

**DOI:** 10.3906/kim-2012-48

**Published:** 2021-06-30

**Authors:** Nurcan KARACA

**Affiliations:** 1 Central Research Laboratory Research and Application Center, Yalova University, Yalova Turkey

**Keywords:** Silica nanoparticles, nanocomposites, photopolymerization, thiol-ene, (3-mercaptopropyl) trimethoxysilane

## Abstract

This article describes the synthesis of modified silica nanoparticles (SiO_2_-MPTMS) via the condensation reaction carried out between silanol moieties of silica nanoparticles and the trialkoxy silyl groups of (3-mercaptopropyl) trimethoxysilane (MPTMS). Then, SiO_2_-MPTMS nanoparticles in certain amounts (0.5 wt %, 1 wt %, 2.5 wt % and 5 wt %) were incorporated into thiol-ene resins consisting of bisphenol A glycerolate dimethacrylate and trimethylolpropane tris(3-mercaptopropionate) to prepare nanocomposite films via the photoinitiated thiol-ene polymerization in presence of 2,2-Dimethoxy-2-phenylacetophenone 99% as a photoinitiator. Fourier transform infrared spectroscopy, dynamic light scattering, scanning transmission electron microscopy, thermal gravimetric analyzer, and X-ray photoelectron spectrometer were employed to characterize SiO_2_-MPTMS nanoparticles. It was revealed that the nanosilica surface was successfully grafted by MPTMS with the grafting ratio of 22.9%. Properties of the nanocomposite films such as decomposition temperature, thermal glass transition temperature, tensile strength, hardness, and particle distribution were investigated and the results were compared with each other and neat film. The addition of MPTMS-modified silica particles did not improve the thermal stability of the films. In scanning electron microscopy study, it was seen that 2.5 wt % of these nanoparticles used as additives were about 200 nm in size and dispersed homogeneously in the polymer matrix. The increase in tensile strength of nanocomposite films compared to the neat film was measured as 77.3% maximum.

## 1. Introduction

In recent years, photoinitiated ‘thiol-ene polymerization’ has recently drawn a great deal of attention in the architecture of advanced polymers due to its advantages such as the rapid polymerization reaction with high rate conversion, insensivity to oxygen and negligible side product formation, leading the typical characteristics of click chemistry [1–4]. This highly efficient click reaction is able to be initiated by UV light irradiation and proceeds via the addition of a thiyl radical across the C=C bond followed by a chain transfer reaction with additional thiol affording the corresponding thioether. Photoinitiated thiol-ene polymerization is a promising candidate for many applications in polymer chemistry such as coatings, adhesives, and dental materials [5–7]. Nowadays, the advantages of this process are incorporated into inorganic nanoparticles with photocurable monomers [8–10].

Silica nanoparticles are of great importance in material science as a nanoplatform for the design of functional materials due to their physical and chemical properties. They have been studied in a variety of fields such as nanohybrids, fillers, drug delivery systems, coupling agents, sensors, and electrical and optical devices [11–13]. The use of trialkoxysilanes as a bonding compound between the surface of inorganic silica and the organic matrix allows improving the particle dispersion by increasing the hydrophobicity of surfaces [14–17]. Nanoparticles with suitable functional groups such as thiols on their surface are notable for their ability to undergo thiol-ene reaction, allowing further functionalization without any indicator or metal catalyst. This desirable method of covalently bonding nanoparticles to the polymer matrix prevents the phase separation and particle agglomeration. Moreover, this opportunity contributes greatly to the production of high-performance materials [18–22]. 

In recent decades, most composite materials used in polymer industry contain special resins based on dimethacrylates. Bisphenol A glycerolate dimethacrylate is one of the commonly used dimethacrylate monomers with reinforcing fillers as an epoxy dimethacrylate. The combination of the epoxy resins and methacyrlates has a major effect in polymerization systems due to the contribution of unique abilities of each. Moreover, their chemical structure includes unsaturated bonds which make them suitable for UV curing technology with radical photoinitiators [23–26]. However, the development of these materials is a hot topic for polymer chemistry, where different expectations arise every day. There are some tendencies to convert long chain compounds to resins before curing [27,28]. Otherwise, thiol-ene chemistry is an attractive method for improving these acrylate systems [29].

This article presents the synthesis of polyorganosiloxane nanoparticles with (3-mercaptopropyl) trimethoxysilane (MPTMS) and the preparation of nanocomposite films with these nanoparticles via the photoinitiated thiol-ene polymerization reaction. Although there are very few articles reported on the application of silica nanoparticles in photopolymerized thiol-ene systems up to date, not enough attention is paid to this field [30,31]. In the literature, Chen et al. studied UV curing photopolymerization of thiol-ene acrylates with commercial silica nanoparticles and investigated the thermal stability and chemical resistance of the resulting products [32]. Sowan et al. used functionalized silica nanoparticles as an additional fragmentation chain transfer agent in the thiol-ene resin system [33]. Wu et al. studied the investigation of the mechanical and physical properties of UV-cured coatings which were prepared with epoxy diacrylate, tripropylene glycol diacrylate, and trimethylolpropane triacrylate in presence of the modified silica nanoparticles, involving (3-mercaptopropyl) trimethoxysilane (MPTMS) [22]. Still, despite all these works, the use of MPTMS modified silica nanoparticles in a thiol-ene acrylate formulation that contains a difunctional epoxy dimethacrylate: bisphenol A glycerolate dimethacrylate (Bis-GMA) and an 3-arm thiol acrylate: trimethylolpropane tris(3-mercaptopropionate) (TMPMP) has not been adequately studied. In this study, after the nanoparticles with silica core covered with MPTMS shield were synthesized and then characterized by scanning transmission electron microscopy (STEM), dynamic light scattering (DLS), Fourier transform infrared spectroscopy (FTIR), and X-ray photoelectron spectrometer (XPS), and the photocured nanocomposite films including these nanoparticles were prepared with bis-GMA and TMPMP. The aim of this study is to reveal the physical and morphological properties of films prepared with MPTMS-modified nanoparticle structures as a nanoplatform by photoinitiated thiol-ene polymerization.

## 2. Results and discussions

### 2.1. Characterization of silica nanoparticles

**Scheme 1:**
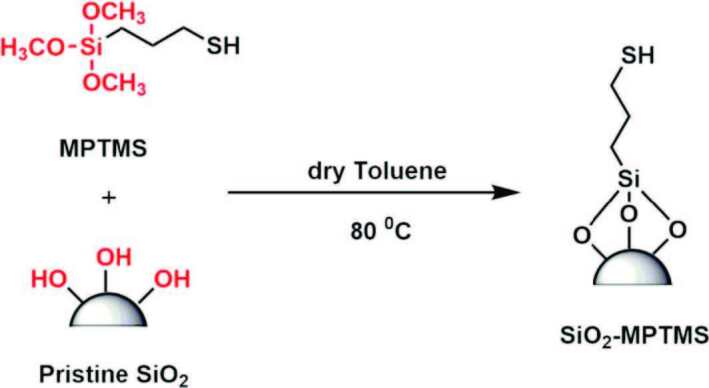
Preparation of MPTMS-modified silica (SiO2-MPTMS) nanoparticles.

**Figure 1:**
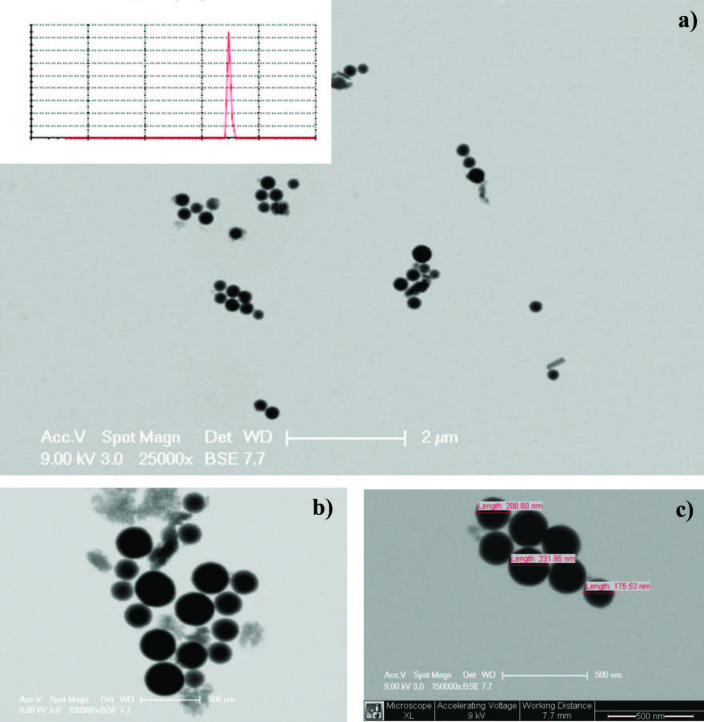
STEM picture of SiO2-MPTMS nanoparticles at a) 25,000  magnification (Inset: DLS spectrum of SiO2-MPTMS nanoparticles in ethanol) b) 100,000  magnification, and c) 150,000  magnification.

Silica nanoparticles with thiol groups (SiO_2_-MPTMS) were synthesized with (3-mercaptopropyl) trimethoxysilane (as shown in Scheme 1) and characterized by determining particle size distribution and morphology using DLS and STEM. Figure 1 shows that SiO_2_-MPTMS nanoparticles were almost spherical in nanometric size of about 200 nm. Furthermore, the size distribution of nanoparticles is centered on a size of 300 nm according to the data obtained from DLS measurement (as shown in Figure 1a). A little change in the size of nanoparticles was observed between the DLS data and STEM images for the reason that the hydrodynamic radiulus of nanoparticles. Scheme 1, Figure 1.

**Figure 2:**
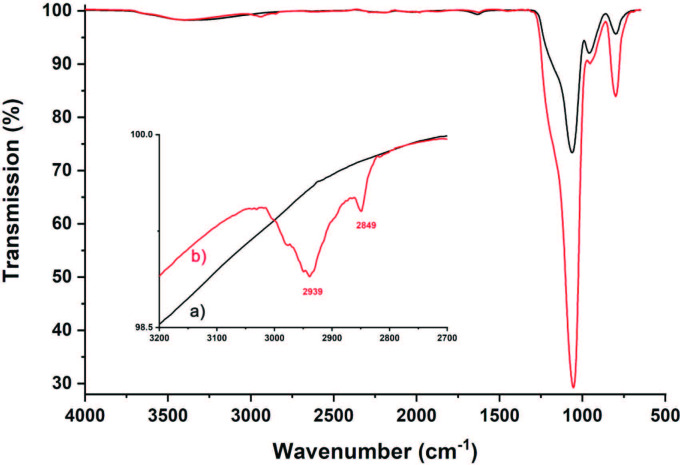
FTIR spectra of a) pristine SiO2 and b) SiO2-MPTMS nanoparticles.

Figure 2 shows the comparison of FTIR spectrum of the pristine and MPTMS-modified SiO_2_ nanoparticles to investigate before and after surface functionalization. The spectra of pristine SiO_2 _(curve a) showed the characteristic stretching and bending vibrations of Si-O-Si and C-Si at 1063 cm^−1^ and 800 cm^−1^, respectively. Moreover, the peak at 960 cm^−1^ indicated the silanol groups (Si-OH streching). At 3385 cm^−1^ and 1637 cm^−1^, O–H stretching and H–O–H bending peaks, respectively, were observed due to the presence of physically adsorbed water on silica samples [27,34]. The spectra of SiO_2_-MPTMS nanoparticles showed additional absorption peaks appearing at 2939 cm^–1^ and 2849 cm^–1^, which are attributed to the aliphatic structure of propyl groups (curve b). The strength of the peaks around 3400 cm^–1^ and 1600 cm^–1^ associated with the stretching of the hydroxyl groups (-OH) on the nanosilica surface almost disappeared, while the 1054 cm^–1^ peak corresponding to Si-O-Si increased. The FTIR spectrum results confirmed that the nanosilica surface was successfully modified by MPTMS silane coupling agent. However, it was seen that the appearance of the peak at 3400 cm^–1^ in curve b was very weak. The condensation reaction carried out between the surface of silica (silanol groups) and MPTMS (-OCH_3_) did not completely consume all -OH groups of the reactants. The peaks of thiol groups (-SH) of MPTMS structures could not be clearly observed in the spectra because of the vibrations overlapping with the vibrations of nanosilica (Figure 2).

**Figure 3:**
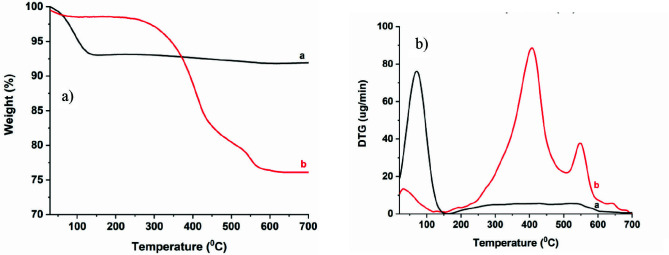
TGA and DTG plots of silica nanoparticles in Figures 3a and 3b, respectively; (a) pristine SiO2 (b) SiO2-MPTMS nanoparticles.

Figure 3 shows the TGA results of the pristine and MPTMS-modified SiO_2_ nanoparticles together with the DTG spectra containing the derivative curves. The thermogram of the unmodified SiO_2_ nanoparticles exhibited a total weight loss of 8.2% (as shown in Figure 3a, curve a). It is known that dehydration of water physically adsorbed from the surface of the silica particles occurs below 120 °C and is followed by dehydroxylation of silanols between 200 °C and 600 °C and above 600 °C [35]. In the TGA spectrum of the SiO_2_-MPTMS nanoparticles, where the mass loss rates observed at the three step of DTG showed the loss up to 120 °C was only 1.4% (as shown in Figures 3a and 3b, curves b). The modification with silane coupling agent increases hydrophobicity which provides protection from the physical storage of the sample under environmental conditions such as water and residual organic solvents [16]. In addition, there are two weight loss stages between 120 °C and 650 °C, 17.6% and 5.1%, respectively, due to the decomposition and the carbonization of the organic part (as shown in Figure 3a, curve b). Indeed, the grafting density of SiO_2_-MPTMS nanoparticles was calculated as 22.9% by comparing the thermogravimetric behavior of the pristine SiO_2_ particles (Figure 3).

**Figure 4:**
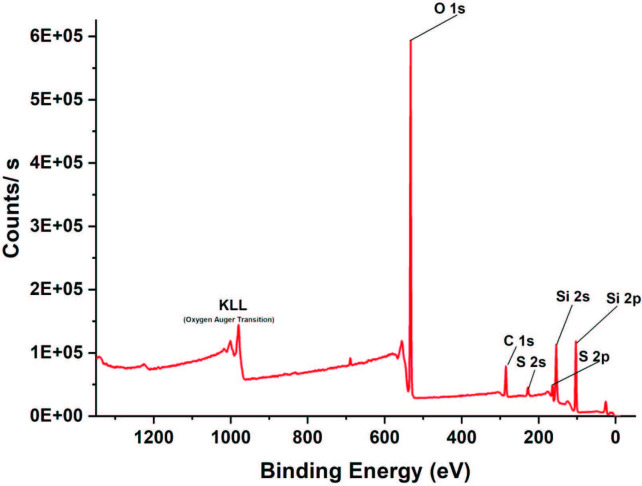
XPS spectra of SiO2-MPTMS nanoparticles.

Further characterization of the SiO_2_-MPTMS nanoparticles was carried out with XPS (as shown in Figure 4). The attachment of MPTMS was clearly confirmed by XPS spectra indicating the presence of groups specific to its chemical composition. While the survey scan of the SiO_2_-MPTMS nanoparticles showed peaks corresponding to O 1s (532.8 eV) and Si 2p (103.8 eV) associated with silica surface, C 1s signal at 286.8 eV and S 2p signal at 164.8 were also observed due to the chemical modification of organic structure having propyl and thiol groups, respectively (Figure 4).

### 2.2. Characterization of nanocomposite films

**Scheme 2:**
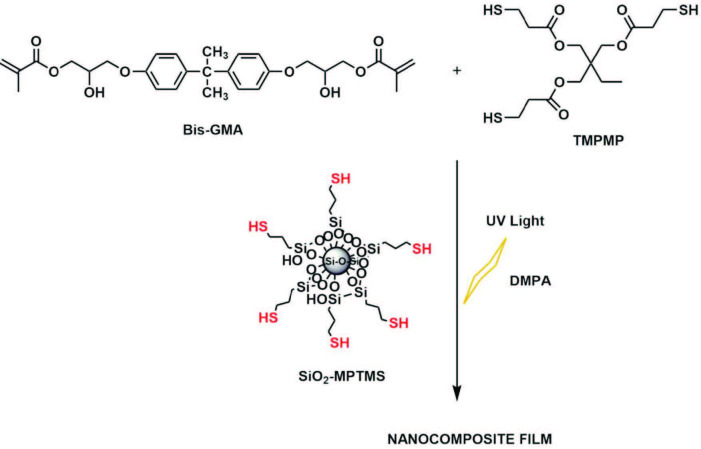
Preparation of nanocomposite films.

Photocured nanocomposite films were prepared with the formulations mixing the selected amounts of SiO_2_-MPTMS (0.5 wt %, 1 wt %, 2.5 wt %, and 5 wt %) with a thiol-ene acrylate system in an equal molar double bond concentration of TMPMP and bis-GMA (as shown in Table 1 and Scheme 2). All cured nanocomposite films showed higher gel content values than the neat formulation (FTE0), indicating the absence of any extractable oligomers or unreacted monomers. The slight differences of gel content percentages of neat and nanocomposite films are attributed to the contribution of the thiol functional groups of MPTMS modified silica nanoparticles to the polymerization conversion. Gel content reveals the consistency of the double bond conversion and also the chemical crosslink density [36] (Scheme 2, Table 1).

**Table 1:** Composition of the thiol-ene resin/silica nanoparticle formulations.

Samples	Molar ratioTMPMP: Bis-GMA (mL/mol)	SiO2-MPTMS nanoparticles (%)	Gel content(%)
FTE0	1:1	0	87.70
FTE0.5MS	1:1	0.5	89.98
FTE1MS	1:1	1	91.56
FTE2.5MS	1:1	2.5	90.08
FTE5MS	1:1	5	88.53

**Figure 5:**
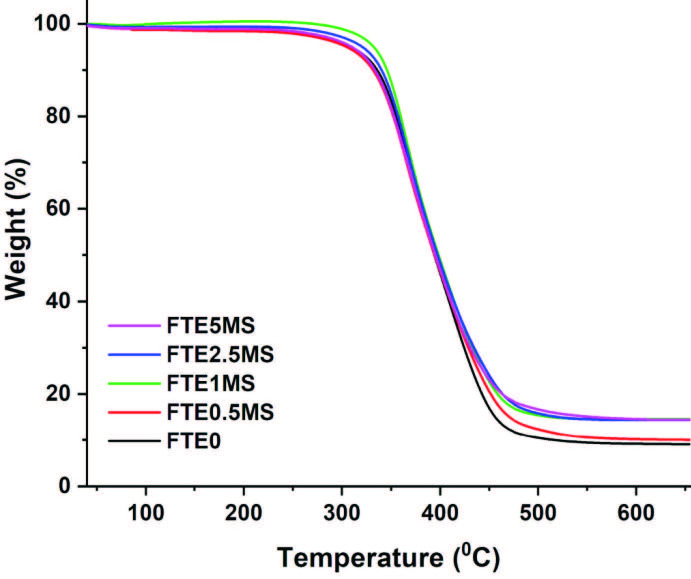
TGA thermograms of SiO2-MPTMS nanocomposite films.

Thermal stability of the nanocomposite films were investigated by TGA. Figure 5 shows the thermogravimetric curves of neat (FTE0) and nanocomposite (FTE0.5MS, FTE1MS, FTE2.5MS, and FTE5MS) films and also, the results are listed in Table 2. The TGA thermograms which were observed in one characteristic weight loss 10% of which was over 330 °C, extended up to 500 °C because of the cleavage of polymer chains. Both neat and hybrid films had close decomposition temperatures. Therefore, it is easy to say that the thermal stability of materials was close to each other. It is known that silica nanoparticles do not significantly change the thermal degradation mechanism of the films to which they are incorporated [18,37]. The chair yields of nanocomposites were higher than the neat film due to the particle content (Table 2, Figure 5).

**Table 2:** Thermal analysis results of SiO2-MPTMS nanocomposite films.

Samples	10% weight-loss temperature (°C)	50% weight-loss temperature (°C)	Max. weight-loss temperature(°C)	Residual part(%)	Glass transition temperature (Tg, °C)
FTE0	335	393.5	365	9.10	139.1
FTE0.5MS	332	394.5	362	10.05	139.2
FTE1MS	345	397.5	364	14.60	139.6
FTE2.5MS	341.5	398.5	367	14.75	139
FTE5MS	334	396	366	14.40	139.2

**Figure 6:**
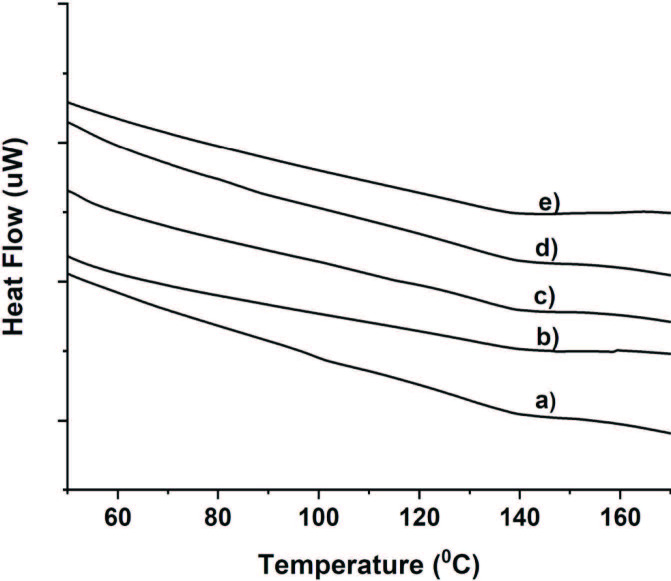
DSC thermograms of SiO2-MPTMS nanocomposite films: a) FTE0, b) FTE0.5MS, c) FTE1MS, d) FTE2.5MS, e) FTE5MS.

Differential scanning calorimetry (DSC) thermograms of neat and hybrid films are shown in Figure 6. Glass transition temperatures (Tg) of nanocomposite films were determined around 138–139 °C (as shown in Table 2). The presence of silica nanoparticles in the polymer matrix did not significantly affect the thermal transition of the films (Figure 6).

**Figure 7:**
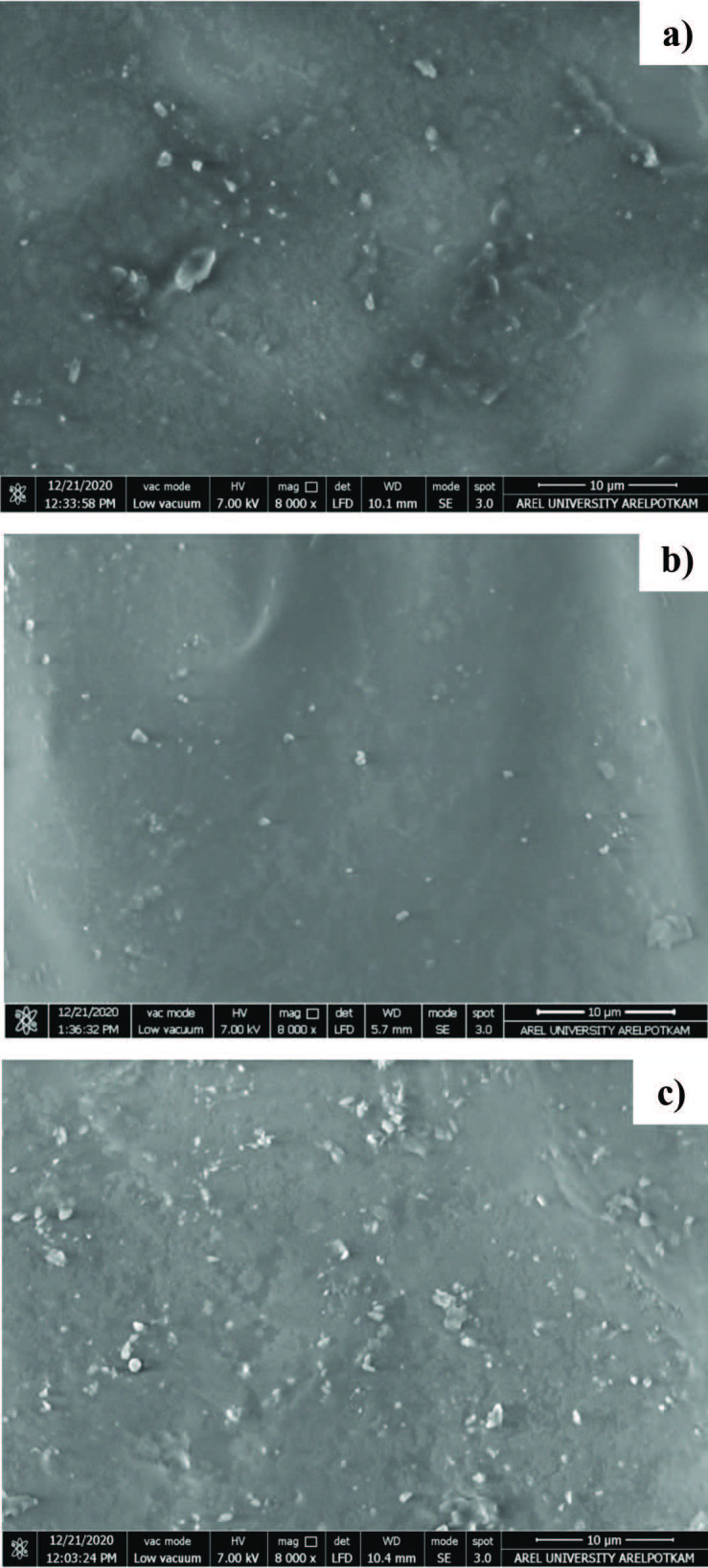
SEM images of SiO2-MPTMS nanocomposite films at 8000  magnification a) FTE0.5MS, b) FTE2.5MS, and c) FTE5MS.

**Figure 8:**
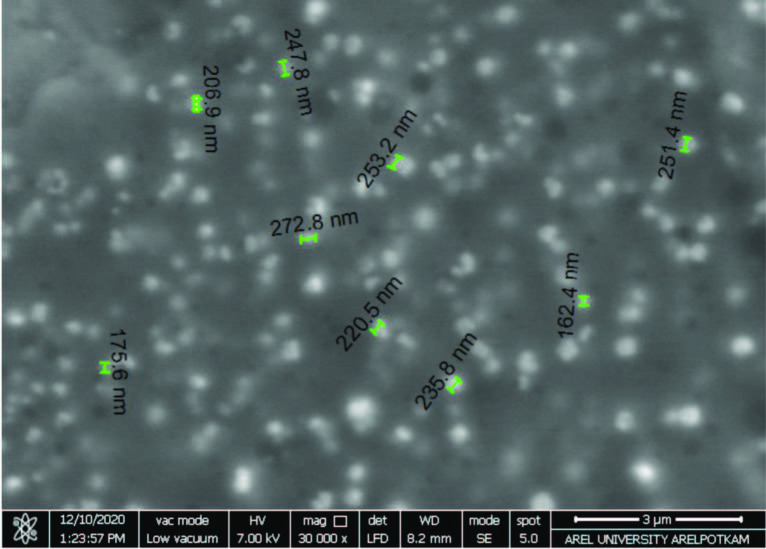
SEM image of MPTMS-SiO2 nanocomposite FTE2.5MS film at 30,000  magnification.

In order to investigate the distribution of SiO_2_-MPTMS nanoparticles in polymer matrices, the morphology of photocured nanocomposite films with certain formulations containing the different particle amounts (0.5 wt %, 2.5 wt %, and 5 wt %) were observed by SEM. The increase in the amount of particles could be confirmed by SEM images of the fracture surfaces of nanocomposite films according to formulation system (as shown in Figure 7). These nanoparticles with different ratios were fine-distributed in the film matrix. However, the aggregations could not be avoided when the addition amount further increased (as shown in Figure 7c). Moreover, a SEM image of the nanocomposite film containing 2.5 wt % particle content was obtained with the cross section of the surface at higher magnification. It was obviously seen that the silica nanoparticles dispersed in the film matrix were a diameter of about 200 nm in a circle appearance (as shown in Figure 8) (Figures 7 and 8).

**Figure 9:**
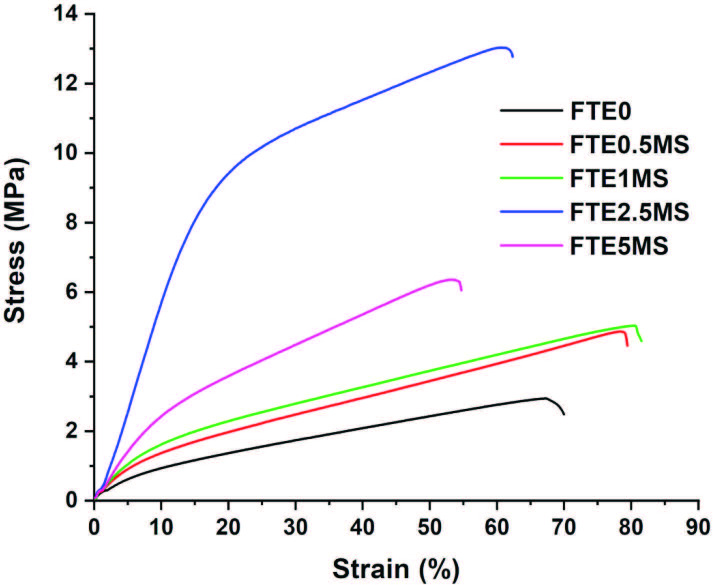
Stress (%)–strain (MPa) curves of SiO2-MPTMS nanocomposite films.

The mechanical properties of prepared nanocomposite films were examined via tensile testing. The stress-strain curves of neat and hybrid films are shown in Figure 9 and the results showing the values of the tensil strength (σ, MPa), elongation at break (ε, %), and young modulus (E, MPa) are summarized in Table 3. The addition of nanoparticles made a significant increase in modulus according to neat film (FTE0) due to the uniform distribution and a good interfacial adhesion in hybrid film matrix. The well dispersed nanoparticles can directly enhance or improve the properties of composite films such as modulus. Moreover, the film flexibility is maintained in the polymer matrix containing nanofillers that have good interaction with the phases because the small sizes of particles do not create large stress [38]. By increasing the SiO_2_-MPTMS nanoparticles content up to 2.5 wt %, the tensile strength increased to 10.06 MPa by 77.3% compared to neat sample (σ_FTE0(MPa) _= 2.94). Efficient hybrid interactions with homogeneous distribution of MPTMS-modified particles allowed to a significant increase in tensile strength. However, with the further addition of silica nanoparticles (5 wt %), the tensile strength and modulus values of the nanocomposite films decreased. In a majority of nanoparticles with more than a certain limit led to the agglomeration due to their heterogeneous dispersion in the film matrix. In addition, the maximum elongation at break of the films was detected in FTE1MS film. According to Shore A hardness tests performed on nanocomposite films, the hardness values showed an increase by the addition of SiO_2_-MPTMS nanoparticles (as shown in Table 3). The maximum Shore A hardness value of the FTE2.5MS film (similarly the tensile testing) was measured as 96 due to the effect of homogeneous sol-gel unit (Table 3, Figure 9). 

**Table 3:** Mechanical properties of SiO2-MPTMS nanocomposite films.

Samples	Tensile strength(MPa)	Elongation at break(%)	Elastic modulus(MPa)	Hardness (Shore A)
FTE0	9.64	70	2.94	80
FTE0.5MS	14.5	80	4.86	86
FTE1MS	21.1	82	5.04	87
FTE2.5MS	61.6	62	13.0	96
FTE5MS	29.7	55	6.36	90

## 3. Conclusion

The SiO_2_-MPTMS nanoparticles were successfully synthesized by a convenient condensation reaction. These nanoparticles were characterized by DLS, STEM, FTIR, and XPS, revealing the size and size distribution, morphology, and functional groups on the surface. The TGA results showed that the grafting ratio of thiol groups was 22.9% pointing to the successful modification process. A series of nanocomposite films were prepared by photoinitiated thiol-ene polymerization of an equimolar TMPMP: bis-GMA. The modulus and tensile strength of nanocomposite films increased as the amount of particles in the polymer matrix increased up to 2.5 wt %. Both STEM and SEM studies showed that the particle sizes of SiO_2_-MPTMS were in the nanoscale. The MPTMS-modified nanoparticles are promising candidates for thiol-ene matrix as a nanofiller.

## 4. Materials and methods

### 4.1. Materials

Tetraethyl orthosilicate 98% (TEOS), (3-mercaptopropyl) trimethoxy silane 95% (MPTMS), bisphenol A glycerolate dimethacrylate (Bis-GMA) Trimethylolpropane tris(3-mercaptopropionate) ≥95% (TMPMP), 2,2-Dimethoxy-2-phenylacetophenone 99% (DMPA) were all purchased from Sigma-Aldrich. Ammonia solution (25%) and all solvents were purchased from Merck. All of the chemical reagents were used as received.

### 4.2. Preparation of silica nanoparticles

The silica nanoparticles were synthesized according to the Stöber method [14,39]. In a 250-mL single-neck round bottom flask, 100 mL of methanol was mixed with 68 mg (1 mmol) of 25%–30% ammonia and 1.98 g (110 mmol) of water, and stirred for 10 min. Then, 10.41 g (50 mmol) of TEOS was added dropwise and the solution was stirred for 3 days. The particles were precipitated in 150 mL of hexane and diethyl ether solution (2:1). It was then isolated and washed three times with ethanol by centrifugation at 4000 rpm for 20 min, and 3.7 g colorless powder is obtained after vacuum.

### 4.3. Surface modification of SiO2 nanoparticles

Five grams of silica particles was dispersed into 300 mL dry toluene in a 500-mL two-necked flask on ultrasonic bath. The reaction flask was equipped with a dropping funnel and condenser on a magnetic stirrer. Ten grams of silane compound agent MPTMS was added dropwise for half an hour at room temperature. After the reaction mixture was stirred for overnight at 80 °C, the particles were isolated via centrifugation at 4000 rpm for 20 min and washed several times with toluene and ethanol to remove the nonbonded MPTMS. Lastly, the synthesized SiO_2_-MPTMS nanoparticles were dried at 90 °C under vacuum for 12 h.

### 4.4. Preparation of nanocomposite coatings

The formulations were prepared by combining the SiO_2_-MPTMS nanoparticles (0.5%, 1%, 2.5%, and 5% by weight of the resin) dispersed in acetone (5 mL) by ultrasonication for 10 min with the mixed thiol-ene resin consisting of bis-GMA and TMPMP in a stoichiometric ratio of 1:1, along with DMPA (0.5% by weight of the resin; as a type I photoinitiator has an absorption extending up to 380 nm [40]) in a 10-mL beaker. The formulations were stirred adequately in an ultrasonic bath and then on a magnetic stirrer at room temperature to obtain homogeneous mixtures, as the solvent was removed by evaporation. The formulation mixtures were then poured onto glass plates and coated onto the surfaces with a gauged bar applicator to obtain uniform thickness of 100 µm. Then, photopolymerization was carried out with a 300 W high pressure mercury lamp (Osram Ultra Vitalux 300 W 230 V E27 UVA/UVB, λmax = 365 nm) for 60 min of irradiation time. The lamp system, where the irradiation source was placed 80 cm above the fixed plate, was cooled by a fan cooling unit. 

Moreover, free films were prepared by pouring into a silicone mold (10 mm × 100 mm × 3.85 mm) with the same formulations with those used in the previous process. After the irradiation (60 min), free nanocomposite films were obtained. 

Additionally, neat films without any nanoparticles as both a coating and a free film were prepared to compare the thermal and mechanical properties with the nanocomposite films. 

The obtained photocured films were dried under vacuum at room temperature. Detailed formulation compositions of the films are listed in Table 1. The characterization of these films was studied with films coated on glass plates. Mechanical tests and SEM analyses were carried out with free films.

### 4.5. Instrumentation

FTIR analysis was performed to characterize the particles on a Perkin-Elmer 100 FTIR spectrometer. The spectra were obtained over the frequency range of 4000–650 cm^–1^ at a resolution of 4 cm^–1^ with an attenuated total reflectance (ATR) cell equipped with a ZnSe single crystal.

DLS measurements were conducted with dilute dispersions of nanoparticles (around 0.1 mg/mL) to reveal their size distributions at 25 °C using Nano-S Zetasizer (Malvern Instruments). 

STEM analysis was carried out for the morphologies of SiO_2_-MPTMS nanoparticles at 25,000×, 100,000×, and 150,000× magnification using Philips-FEI XL30 ESEM-FEG SEM.

XPS was used to characterize the modification effects of nanoparticles using a Thermo Scientific K-Alpha XPS with Al K α X-ray radiation with energy of 150.0 eV and take off angle of 90°. The data acquired was shifted in reference to C 1s quantum level positioned at 254.8 eV.

The thermogravimetric analysis (TGA) was performed in a SEIKO TG/DTA 6300 analyzer using alumina sample pan under nitrogen atmosphere (50 mL/min) from room temperature to 650 °C with a heating rate of 10 °C/min. The grafting ratio calculated according to the Eq. (1) [22].

R *_g_* =(W_1_/W’_1_ -W_0_/W’_0_ )×100% (1)

W_1_ is the starting weight of the modiﬁed silica nanoparticles and W’_1_ is the residual weight of the modiﬁed silica nanoparticles at 650 °C; W_0_ is the starting weight of unmodiﬁed silica nanoparticles, and W_0_’ is the residual weight of unmodiﬁed silica nanoparticles at 650 °C.

The gel content was determined on the neat and nanocomposite films by measuring the weight loss after 20 h extraction with boiling acetone according to the standard test method (ASTM D2765).

The morphology of the nanocomposite films was investigated by SEM using an FEI QUANTA 450 FEG ESEM SEM FEG 450 under low vacuum. SEM images of the fracture surfaces and the cross section of nanocomposite films were taken at 8000× and 30,000× magnification, respectively.

The thermal characterization of the films was performed by DSC using Seiko DSC 7020 system. Approximately 10 mg of the composite films was put in aluminum pan and heated at a 0–250 °C temperature range with a rate of 10 °C/ min under nitrogen atmosphere (50 mL/min).

Mechanical properties of free ﬁlm sheets were investigated using a Zwick/Roell Z1.0 universal test machine. Thus, specimens (length: 100 mm; width: 9.5 mm; thickness: 3.5 mm, on average) were tested at room temperature according to the DIN EN ISO 527-1 standard with a crosshead speed of 50 mm/min. Moreover, hardness of free ﬁlms was measured in Shore A scale using a Zwick hardness tester (Zwick, Ulm, Germany) according to ASTM D-2240.
